# A compact five-gene immuno-fibrotic profile distinguishing systemic sclerosis subtypes with PXDN validated as a fibrosis-associated target

**DOI:** 10.3389/fimmu.2026.1833652

**Published:** 2026-08-19

**Authors:** Xiangyue Zhao, Kejian Hu, Yinzhi Cui, Wen Li, Zhiyao Li, Pu Feng, Wenping Xie, Xingcheng Xiang, Wen Xu, Junzhang Chen, Pingcuo Wangzha, Xiao Long, Jiucun Wang, Haiyan Chu

**Affiliations:** 1 Department of Plastic Surgery, Peking Union Medical College Hospital, Chinese Academy of Medical Sciences & Peking Union Medical College, Beijing, China; 2 Research Unit of Dissecting the Population Genetics and Developing New Technologies for Treatment and Prevention of Skin Phenotypes and Dermatological Diseases (2019RU058), Chinese Academy of Medical Sciences, Shanghai, China; 3 Department of Laboratory Medicine, Zhongshan Hospital, Fudan University, Shanghai, China; 4 State Key Laboratory of Genetics and Development of Complex Phenotypes, School of Life Science & Human Phenome Institute, Fudan University, Shanghai, China; 5 High School Affiliated to Shanghai Jiao Tong University, Shanghai, China; 6 School of Basic Medical Sciences, Peking University, Beijing, China; 7 Division of Dermatology, Huashan Hospital, Fudan University, Shanghai Institute of Dermatology, Shanghai, China; 8 State Key Laboratory of Genetics and Development of Complex Phenotypes, School of Life Science & Shanghai Pudong Hospital, Fudan University, Shanghai, China

**Keywords:** biomarkers, gene signature, immune infiltration, machine learning, PXDN, systemic sclerosis, weighted gene co-expression network analysis (WGCNA)

## Abstract

**Introduction:**

Systemic sclerosis (SSc) is a heterogeneous autoimmune disease characterized by a paucity of reliable biomarkers for accurate diagnosis and subtype stratification. The considerable clinical variability between diffuse and limited cutaneous subtypes underscores an urgent need for molecular tools that can dissect this heterogeneity and guide therapeutic strategies.

**Methods:**

To address this, we employed a multi-step computational approach. Initially, Weighted Gene Co-expression Network Analysis (WGCNA) was applied to the GSE130955 dataset to identify disease-associated modules. This was followed by the application of three machine-learning algorithms—LASSO regression, random forest, and SVM-RFE—to the GSE181549 dataset for hub gene selection. Immune cell infiltration was estimated using CIBERSORT, and the expression of candidate genes was validated at single-cell resolution using the GSE138669 dataset. Experimental validation was performed for the top candidate, *PXDN*, assessing its protein expression in human fibroblasts, SSc patient skin, and a bleomycin-induced murine fibrosis model. Finally, molecular docking with AutoDock Vina was conducted to evaluate the binding affinity of small molecules to *PXDN*.

**Results:**

WGCNA identified a module strongly correlated with SSc status (r = 0.73, p < 2e-16), which was enriched in immune chemotaxis and extracellular matrix organization pathways. The convergence of the three machine-learning algorithms nominated a five-gene signature (*CPXM1, ELN, GSTM5, PXDN*, *PDE7B*), which demonstrated high diagnostic accuracy (AUC = 0.985, 95% CI: 0.965-1) and effectively distinguished diffuse from limited cutaneous SSc. Single-cell analysis confirmed predominant expression of these genes in fibroblast and macrophage populations within SSc lesional skin. Experimentally, *PXDN* was found to be upregulated by TGF-β1 in human fibroblasts and was significantly elevated in skin and lung tissues from SSc and IPF patients, as well as in the bleomycin-induced mouse model. Although molecular docking nominated Protokylol hydrochloride as a compound satisfying distal-cavity geometric criteria, this serves as a hypothesis-generating finding rather than a confirmed inhibitor.

**Discussion:**

Our integrative analysis identifies a concise immunofibrotic gene signature that robustly distinguishes SSc and its major subtypes. This signature highlights a potential nexus of immune-stromal interactions that may underlie disease heterogeneity, offering candidate biomarkers for molecular stratification and therapeutic targeting. Notably, *PXDN* emerges as a particularly promising target, warranting further experimental investigation to validate its functional role and therapeutic potential in SSc.

## 1 Introduction

Systemic Sclerosis (SSc) is a highly heterogeneous autoimmune connective tissue disease, characterized by the triad of vascular dysfunction, immune dysregulation, and progressive fibrosis affecting skin and internal organs (1). Its clinical presentation, natural history, and prognosis vary dramatically among individuals, presenting a central chanllenge to both clinical management and pathogenic research.

Clinically, patients are categorized into limited (lcSSc) and diffuse cutaneous systemic sclerosis (dcSSc) subsets based on skin involvement extent (2). lcSSc is characterized by fibrosis confined to the fingers (sclerodactyly), distal extremities and face, often preceded by a long history of Raynaud phenomenon. Whereas dcSSc features rapid, widespread skin thickening extending to the trunk and proximal extremities, and is associated with early and severe visceral organ involvement (3). Pulmonary fibrosis is the main visceral involvement characteristic of SSc. This clinical phenotypic divergence translates into significant survival differences, with 5-year-survival of 84% in dcSSc and 93% to 96% in lcSSc (4). Prognostic variability is further compounded by factors such as sex, age, organ manifestations, specific autoantibody profiles (5). SSc has one of the highest mortality rates among connective tissue diseases (CTD) (6). The leading causes of mortality, interstitial lung disease, pulmonary artery hypertension (PAH), and cardiac involvement further underscore the variable organ tropism of the disease (7).

Overall, this pronounced clinical heterogeneity underlies divergent prognoses and treatment responses, complicating management and hindering therapeutic development. Current clinical classification lacks mechanistic resolution and molecular markers that reliably stratify patients or predict trajectory. It necessitates a paradigm shift from descriptive classification to mechanistic stratification. Merely cataloging symptoms is insufficient; the future lies in deconvoluting this complexity through the integration of multi-omics data.

High-throughput transcriptomics and single-cell profiling have uncovered candidate pathways and cell populations that contribute to SSc pathogenesis (8–10). However, single-method analyses can produce large candidate lists and variable signatures across cohorts, limiting clinical translation. To address these limitations, integrative approaches that combine systems-level network analysis with robust feature selection are needed.

Weighted Gene Correlation Network Analysis (WGCNA) groups genes into biologically coherent modules and links modules to clinical traits, efficiently identify functionally relevant gene sets (11, 12). Nonetheless, WGCNA modules typically contain a large number of genes, so subsequent refinement is required to extract compact, predictive biomarkers. Machine learning (ML) algorithms feature selection methods (LASSO, random forest, SVM-RFE) excel at reducing dimensionality and selecting parsimonious gene panels with high discriminative power (13, 14). Integrating WGCNA with multiple machine-learning algorithms leverages complementary strengths: network-level biological context and robust predictive selection.

In this study, we applied WGCNA to early SSc skin transcriptomes (GSE130955) to identify disease-associated modules, then refined hub genes using three machine-learning algorithms on a larger cohort (GSE181549). We assessed immune correlates via CIBERSORT and validated cellular sources using single-cell RNA-seq (GSE138669). Our integrated pipeline yielded a five-gene immunofibrotic signature (*PXDN, CPXM1, ELN, PDE7B, GSTM5*) that accurately distinguishes SSc from controls and discriminates major cutaneous subtypes, pointing to macrophage–fibroblast interactions as a driver of disease heterogeneity. Notably, PXDN was further validated as a key factor, with its expression being upregulated both by TGF-β stimulation in human foreskin fibroblasts (HFFs) and in bleomycin-induced mouse lung fibrosis tissues, indicating its central role in fibrotic processes.

Notably, the prioritization of PXDN by our unbiased pipeline is concordant with its well-established biology in fibrosis. PXDN (peroxidasin) is a heme-containing peroxidase induced during TGF-β1-driven myofibroblast differentiation, secreted into the extracellular space, incorporated into the extracellular matrix, and co-localizing with fibronectin (15). Mechanistically, PXDN generates hypobromous acid (HOBr) to catalyze sulfilimine crosslinks between collagen IV molecules, stabilizing basement membranes—an ECM-stiffening process inherently relevant to fibrotic remodeling (16), and is increasingly recognized as a node linking ECM crosslinking, oxidative stress, and tissue remodeling across fibrotic conditions (17). Rather than a novel hit, we therefore regard PXDN as a mechanistically grounded fibrosis node that our integrative pipeline has independently rediscovered and confirmed in SSc skin.

## 2 Materials and methods

### 2.1 Data source

Based on their relatively larger sample sizes and the availability of comprehensive clinical metadata, two independent SSc transcriptomic datasets were selected for this integrated analysis to ensure robust subtype stratification and biomarker identification. The gene expression data used is sourced from the Gene Expression Omnibus (GEO) database of the National Center for Biotechnology Information (NCBI) in the United States, with dataset number GSE130955 (18). Using second-generation RNA sequencing technology, RNA was detected in skin biopsy samples from 48 patients (with an average course of 1.3 years) and 33 matched healthy controls in the Prospective Registry of Early Systemic Sclerosis (PRESS) cohort. GSE181549 (19), A total of 339 forearm skin biopsies were obtained from 113 SSc patients and 44 matched healthy controls. 105 SSc patients had a 2nd biopsy, and 76 had a 3rd biopsy.

We do WGCNA analysis for GSE130955 dataset and do three machine learning methods for GSE181549 dataset, and according to disease subtypes and disease duration we split the dataset into two group and do the learning. Machine Learning Analysis on GSE181549 Dataset. GSE138669 Scleroderma skin single-cell data was used for further validation.

### 2.2 Data preprocessing and weighted gene co-expression network analysis

The R package WGCNA v1.73 (11) was utilized to construct a weighted gene co-expression network. First, sample quality was assessed using the goodSamplesGenes function. Hierarchical clustering was performed on the samples to detect outliers, and samples with a height cut-off greater than 65 were excluded from the analysis to ensure network stability. For data normalization, raw RNA-sequencing counts were normalized using the variance-stabilizing transformation (VST, DESeq2) to obtain log2-scale expression values; cross-dataset batch effects were inspected and, where present, corrected with ComBat (sva package), and low-expression genes were filtered prior to network construction.

To construct the network, we employed the blockwiseModules function. We first determined an appropriate soft-thresholding power to ensure the network approximated a scale-free topology. Based on the scale-free topology criterion, a power of β = 10 was selected automatically. We constructed an adjacency matrix which was transformed into a Topological Overlap Matrix (TOM) to measure the network connectivity of a gene defined as the sum of its adjacency with all other genes for network generation, setting the TOM type to “unsigned”.

Gene modules were identified using average linkage hierarchical clustering based on the TOM-based dissimilarity measure. We imposed a minimum module size of 30 genes (minModuleSize = 30). To merge highly similar modules, a cut height of 0.25 (mergeCutHeight = 0.25) was applied, corresponding to a correlation threshold of 0.75.

### 2.3 Identification of clinically significant modules

To identify modules significantly associated with Systemic Sclerosis (SSc), we utilized Module Eigengenes (MEs), which represent the first principal component of a given module and summarize its gene expression profile. Pearson correlation coefficients were calculated between the MEs and the clinical traits (SSc vs. Control). The statistical significance of these correlations was assessed using Student’s t-test (corPvalueStudent). Modules with high correlation coefficients and significant $p$-values were selected for downstream analysis. Visualization of the module-trait relationships was performed using the labeledHeatmap function.

### 2.4 Identification of hub genes

To screen for key genes within the significant modules, we defined two metrics: Module Membership (MM) and Gene Significance (GS). MM represents the correlation between the gene expression profile and the Module Eigengene, measuring how close a gene is to the module center. GS represents the correlation between the gene and the clinical trait (SSc).

Hub genes were defined as those exhibiting high intramodular connectivity and high clinical significance. We set the selection criteria as |MM| > 0.8 and |GS| > 0.5. These genes were considered biologically relevant candidates driving the module’s association with the disease.

### 2.5 Functional enrichment analysis

To explore the biological functions and signaling pathways associated with the key modules, we performed Gene Ontology (GO) and Kyoto Encyclopedia of Genes and Genomes (KEGG) pathway enrichment analyses using the clusterProfiler R package. The org.Hs.eg.db package was used for gene ID conversion. For GO analysis, we examined three sub-ontologies: Biological Process (BP), Cellular Component (CC), and Molecular Function (MF). Statistical significance was defined using a p-value cutoff of 0.05 and a q-value cutoff of 0.1. The results were visualized using SCP (Single-Cell Pipeline) package.

### 2.6 Machine learning-based marker screening

To further validate and narrow down the hub genes to robust diagnostic markers, we employed three machine learning algorithms: Least Absolute Shrinkage and Selection Operator (LASSO), Random Forest (RF), and Support Vector Machine-Recursive Feature Elimination (SVM-RFE). LASSO regression was implemented using the glmnet package to minimize the potential overfitting, using 10-fold cross-validation. Random Forest was performed using the randomForest package to rank genes based on variable importance. SVM-RFE was conducted using the e1071 package to select the optimal feature subset with the highest classification accuracy. Overlapping genes identified by these algorithms and the WGCNA hub genes were considered potential biomarkers. To minimize overfitting, all models were trained within a repeated cross-validation framework: LASSO logistic regression (glmnet) used 10-fold cross-validation with λ selected at minimum binomial deviance (lambda.min); Random Forest (randomForest) was run with 500 trees and default mtry, ranking genes by Mean Decrease Gini; SVM-RFE (e1071, linear kernel) used 5-fold cross-validation to select the most accurate feature subset. Only genes consistently selected by all three algorithms and overlapping with WGCNA hub genes were retained, and performance was confirmed in an independent dataset to guard against overfitting.

Three distinct stratification approaches were applied to evaluate classification performance under different clinical contexts:

Approach 1 Case-Control Classification (Benchmark): As a benchmark for comparison, a conventional binary classification was performed to differentiate All SSc Cases (n=295) from Healthy Controls (n=44). This approach serves as a reference model for evaluating the performance of the more granular stratification strategies outlined above.

Approach 2 Stratification by Clinical Subtype: Following the exclusion of healthy controls (n=44), patients were classified according to established clinical subtypes: Diffuse Cutaneous Systemic Sclerosis (dcSSc) (n=180) and Limited Cutaneous Systemic Sclerosis (lcSSc) (n=115). Binary classification models were then developed to distinguish between these two major SSc subtypes.

Approach 3 Stratification by Disease Duration: Subjects from the disease cohort (n=295) were stratified into two groups based on the median disease duration of 3.192 years. The Long-Duration group (n=147) consisted of patients with disease duration > 3.2 years, while the Short-Duration group (n=148) included patients with disease duration ≤ 3.2 years. Binary classification models were subsequently trained to discriminate between these two duration-based groups.

### 2.7 Single-cell gene knockdown simulation

scTenifoldKnk is an efficient virtual knockout tool for gene function predictions via single-cell gene regulatory network perturbation (20). The scTenifoldKnk R package (ver 1.0.1) was applied to perform PXDN perturbation, with qc_mtThreshold = 0.1, qc_minLSize = 500, nc_nNet = 15, nc_nCells = 500, nc_nComp = 5 to do the Fibroblast PXDN perturbation.

### 2.8 Cell culture

Human foreskin fibroblasts (HFFs) were cultured in Dulbecco’s Modified Eagle Medium (DMEM) supplemented with 10% fetal bovine serum (FBS) and maintained in a humidified incubator at 37 °C with 5% CO_2_. Cells were passaged at least three times after thawing to ensure stabilization before use in experiments. For treatments, cells were stimulated with 10 ng/mL TGF-β for 48 hours. Following stimulation, total cellular proteins were extracted using RIPA lysis buffer containing phenylmethylsulfonyl fluoride (PMSF).

### 2.9 Mouse lung fibrosis model

A pulmonary fibrosis model was established by intratracheal instillation of bleomycin (BLM) in 6- to 8-week-old mice. The use of 6- to 8-week-old mice follows standard protocols for bleomycin-induced fibrosis models, as this age range represents sexually mature young adults with a fully developed immune system that responds consistently to bleomycin challenge. Control (saline-treated) mice served as the baseline reference group and were processed under identical conditions. Mice received BLM at a dose of 2.5 μg/μL in salin, with the administration volume adjusted according to body weight (e.g., 20 μL for a 20 g mouse). Control mice received an equivalent volume of sterile saline. After 21 days, lung tissues were harvested for analysis.

### 2.10 Antibodies and reagents

Antibodies against alpha Tubulin (ab52866, dilution: 1:1000), FN1 (ab45688, dilution: 1:1000), were from abcam (Boston, MA, USA). α-SMA (GB1011364, dilution: 1:1000), was from Servicebio (Wuhan, China); PXDN (FNab10858, dilution: 1:1000), was from FineTest (Wuhan, China). HRP-conjugated Affinipure Goat Anti-Mouse IgG (GB23301, dilution: 1:10000) and HRP-conjugated Affinipure Goat Anti-Rabbit IgG (GB23303, dilution: 1:10000) were from Servicebio. The recombinant human TGFβ1 protein (HY-P70543) was obtained from MedChemExpress (MCE, Monmouth Junction, NJ, USA). Bleomycin hydrochloride was purchased from Hanhui Pharmaceutical Co., Ltd. (Shanghai, China).

### 2.11 Extraction of proteins and western blot analysis

Whole cell lysates were extracted with RIPA buffer containing PMSF. Protein samples were separated by 10% SDS-PAGE and transferred onto PVDF membrane (Millipore). After incubation with horseradish peroxidase–conjugated secondary antibodies, protein bands were visualized using a chemiluminescence imaging system (Tanon 4600SF, Tanon Science & Technology Co., Ltd., Shanghai, China). Band intensity was quantified by measuring the gray scale of the image with ImageJ.

### 2.12 Immunofluorescence

Sections were deparaffinized, rehydrated, and subjected to heat-induced antigen retrieval in citrate buffer (pH 6.0) for 15 min. After cooling, sections were permeabilized with 0.3% Triton X-100 for 15 min and blocked with 5% normal serum for 1 h at room temperature. Slides were incubated with primary antibody overnight at 4 °C, followed by fluorophore-conjugated secondary antibody for 1 h at room temperature in the dark. Nuclei were stained with DAPI. Slides were mounted with anti-fade medium and imaged.

### 2.13 Molecular docking analysis

The PXDN predicted structure (AF-Q92626-F1) was retrieved from AlphaFold Database (21). The peroxidase domain (residues 698–1265) was extracted. Eosinophil peroxidase (EPO; PDB: 8OGI, 1.55 Å) was selected as the primary template for heme placement due to closer homology (two covalent heme ester bonds, shared with PXDN) than myeloperoxidase (MPO; PDB: 1D2V, 1.75 Å), which was used only for cross-validation (22).

**Figure 1:**
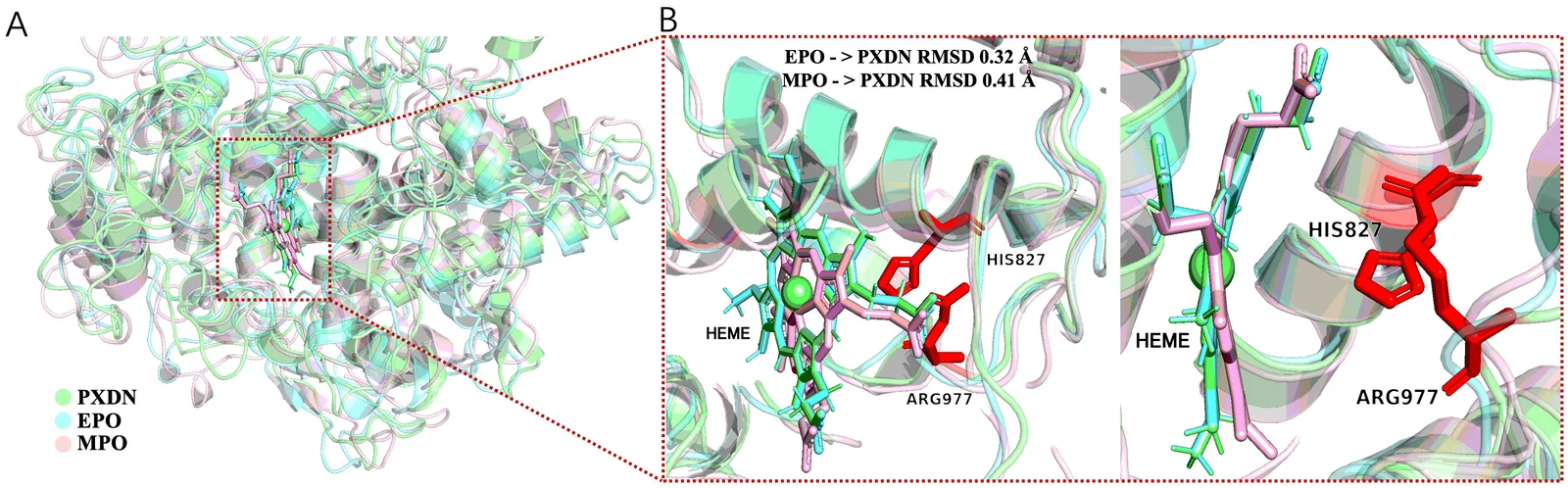
Structural superposition of the PXDN, EPO, and MPO active sites. **(A)** The PXDN peroxidase domain (lime cartoon) is shown with the EPO-derived heme cofactor and heme iron (lime sphere). EPO (palecyan) and MPO (lightpink) catalytic domains were superposed onto PXDN using the conserved active-site triangle (proximal HIS, distal HIS, and proximal ARG Cα atoms). **(B)** The distal histidine (HIS827, red sticks), distal arginine (ARG977, red sticks) of PXDN are labeled. Superposition RMSD values: EPO→PXDN, 0.32 Å; MPO→PXDN, 0.41 Å. The EPO- and MPO-derived heme iron positions in the PXDN frame agree within <1.0 Å.

The distal cavity was defined by the conserved catalytic residues of heme peroxidases (22, 23). The EPO active-site triangle (Cα atoms of proximal HIS B474, distal HIS A233, and proximal ARG B471) was superposed onto PXDN using the Kabsch algorithm. The best-matching PXDN triad (HIS1074–HIS827–ARG1071) gave an RMSD of 0.32 Å. Independent MPO superposition (HIS C336–HIS A95–ARG C333) yielded 0.41 Å; the two heme iron predictions agreed within <1.0 Å. The distal His (HIS827, light-chain-equivalent) and distal Arg (ARG977, the sole arginine within 8 Å of HIS827 on the distal side) were identified as the catalytic pair (**Figure 1**).

Molecular docking used AutoDock Vina (24). The grid center (7.076, −6.636, −3.757) was the midpoint of HIS827 Nϵ2 and ARG977 Cζ, translated along the substrate-channel axis into the cavity interior to enclose the substrate-binding volume rather than the cavity floor (22). The box (12 × 12 × 12 Å) excluded heme Fe (9.6 Å away; **Figure 1A**). Exhaustiveness = 16, num_modes = 10. A commercial FDA-approved drug library containing 2,203 compounds (MedChemExpress, Cat. No. HY-L022, Monmouth Junction, NJ, USA) was used for virtual screening. The docking results were analyzed, and protein–ligand interaction patterns were visualized using UCSF ChimeraX (25).

Docking poses were filtered by three criteria grounded in peroxidase catalytic geometry (22, 23, 26): (i) ligand contact ≤ 3.5 Å to HIS827 Nϵ2 [acid–base H-bond range (22)]; (ii) ≤ 4.0 Å to ARG977 Cζ [guanidinium electrostatic stabilization range (23)]; and (iii) > 4.5 Å to heme Fe, exceeding twice the Fe–ligand coordination radius (26) and confirming distal-side occupancy.

### 2.14 Statistical analysis

All statistical analysis were performed using R software (version 4.4.1). Continuous data were presented as mean ± standard deviation (SD) if they followed a normal distribution. For comparisons between two independent groups that met both normality and homogeneity of variance assumptions, an independent two-sample Student’s t-test was applied. The threshold for statistical significance was set at a two-tailed p-value < 0.05 for all analyses unless otherwise specified. To address potential overfitting, the five-gene signature derived from the GSE181549 discovery cohort was independently validated in the separate GSE130955 cohort, which was not used for feature selection and thus served as an external validation set; AUCs were reported with 95% confidence intervals in both cohorts.

## 3 Results

### 3.1 WGCNA identifies key gene modules correlated with SSc clinical traits

**Figure 2:**
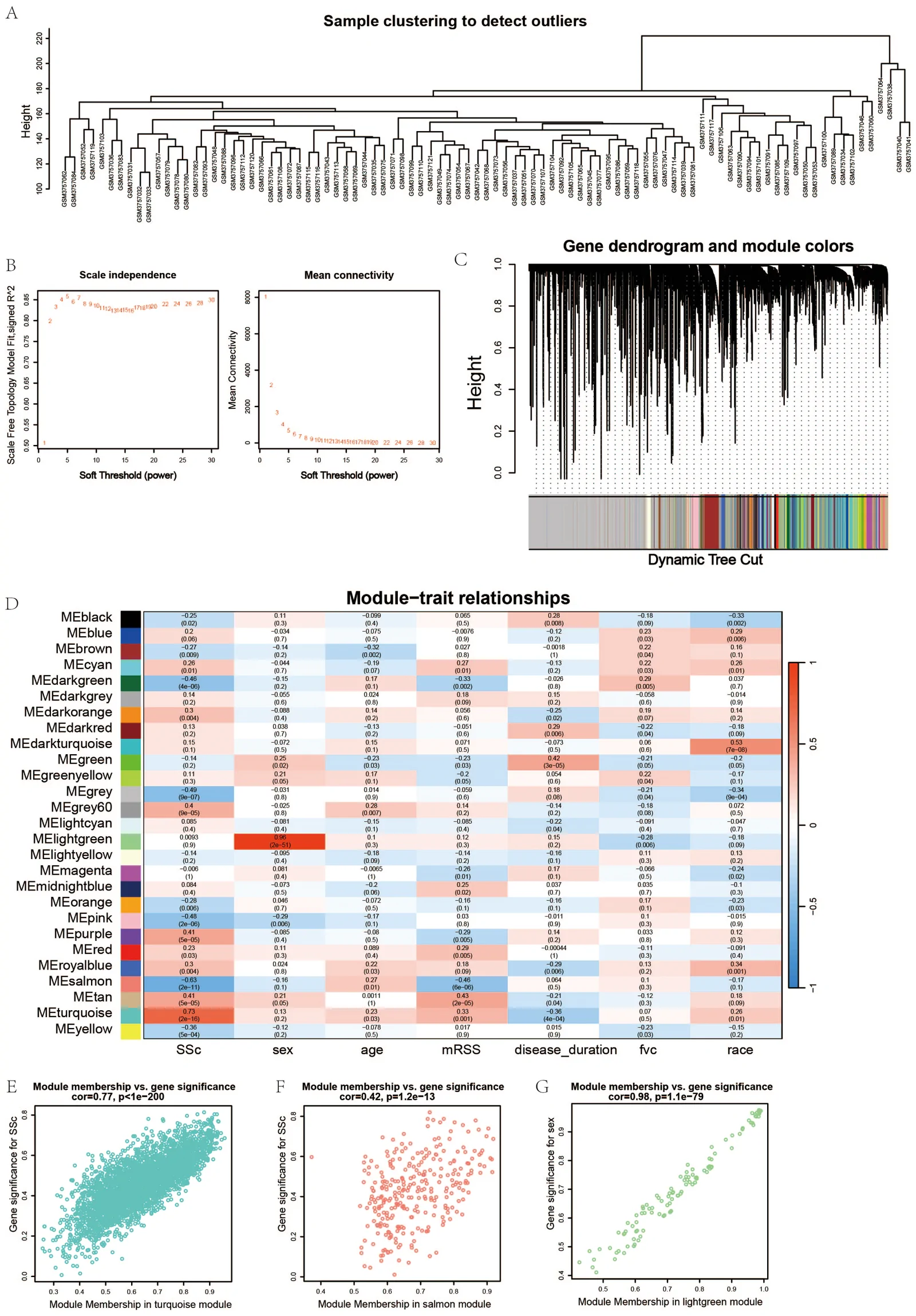
Weighted gene co-expression network analysis (WGCNA) and module–trait associations. **(A)** Sample clustering dendrogram for GSE130955 illustrating sample relationships and quality-control filtering. Samples identified as outliers (cutoff = 200) were removed. **(B)** Determination of the soft-thresholding power: scale-free topology fit index (left) and mean connectivity (right). A power of 10 was chosen to approximate scale-free topology. **(C)** Gene dendrogram from hierarchical clustering based on topological overlap; module assignments were determined by the dynamic tree-cut algorithm. **(D)** Heatmap of correlations between 27 co-expression modules and clinical traits (SSc status, gender, age, skin score, mRSS, disease duration, and FVC). The turquoise module shows the strongest positive correlation with systemic sclerosis (r = 0.73), the lightgreen module is most strongly associated with sex, and the salmon module displays the strongest negative correlation with SSc (r = −0.63). **(E)** Scatter plot of module membership (kME) versus gene significance (GS) for SSc in the turquoise module, showing a strong positive relationship. **(F)** Scatter plot of module membership versus gene significance for SSc in the salmon module. **(G)** Scatter plot of module membership versus gene significance for sex in the lightgreen module, demonstrating a strong positive correlation.

We first performed sample clustering to address clinical heterogeneity in systemic sclerosis (SSc); samples flagged as relative outliers by a cutoff value of 200 were excluded from downstream analyses (**Figure 2A**). For weighted gene co-expression network analysis (WGCNA), a soft-thresholding power of 10 was selected automatically to approximate scale-free topology (**Figure 2B**). Using the dynamic tree-cut algorithm, 12,486 genes were organized into 27 discrete co-expression modules (**Figures 2C, D**).

Within each module, hub genes were defined by eigengene-based connectivity (kME). To assess the disease relevance of modules, we correlated module membership (MM) with gene significance (GS). The Turquoise module showed a particularly strong MM–GS relationship (r = 0.77, p < 1e-200), indicating that genes central to this module are highly associated with the SSc phenotype (**Figure 2E**).

Focusing on modules most tightly linked to SSc traits, MEturquoise (2,381 genes) exhibited a strong positive correlation with SSc disease status (r = 0.73, p = 2e-16) and positive associations with measures of skin involvement, including skin score and modified Rodnan skin score (mRSS), suggesting this module captures biology related to disease presence and severity. MEsalmon, in contrast, showed a significant negative correlation with SSc status (r = −0.63, p = 2e-11) and inverse correlations with skin score and mRSS, consistent with a module whose higher expression may be associated with milder disease or remission. MEturquoise displayed a weak, non-significant negative correlation with disease duration, a result that warrants further investigation.

**Figure 3:**
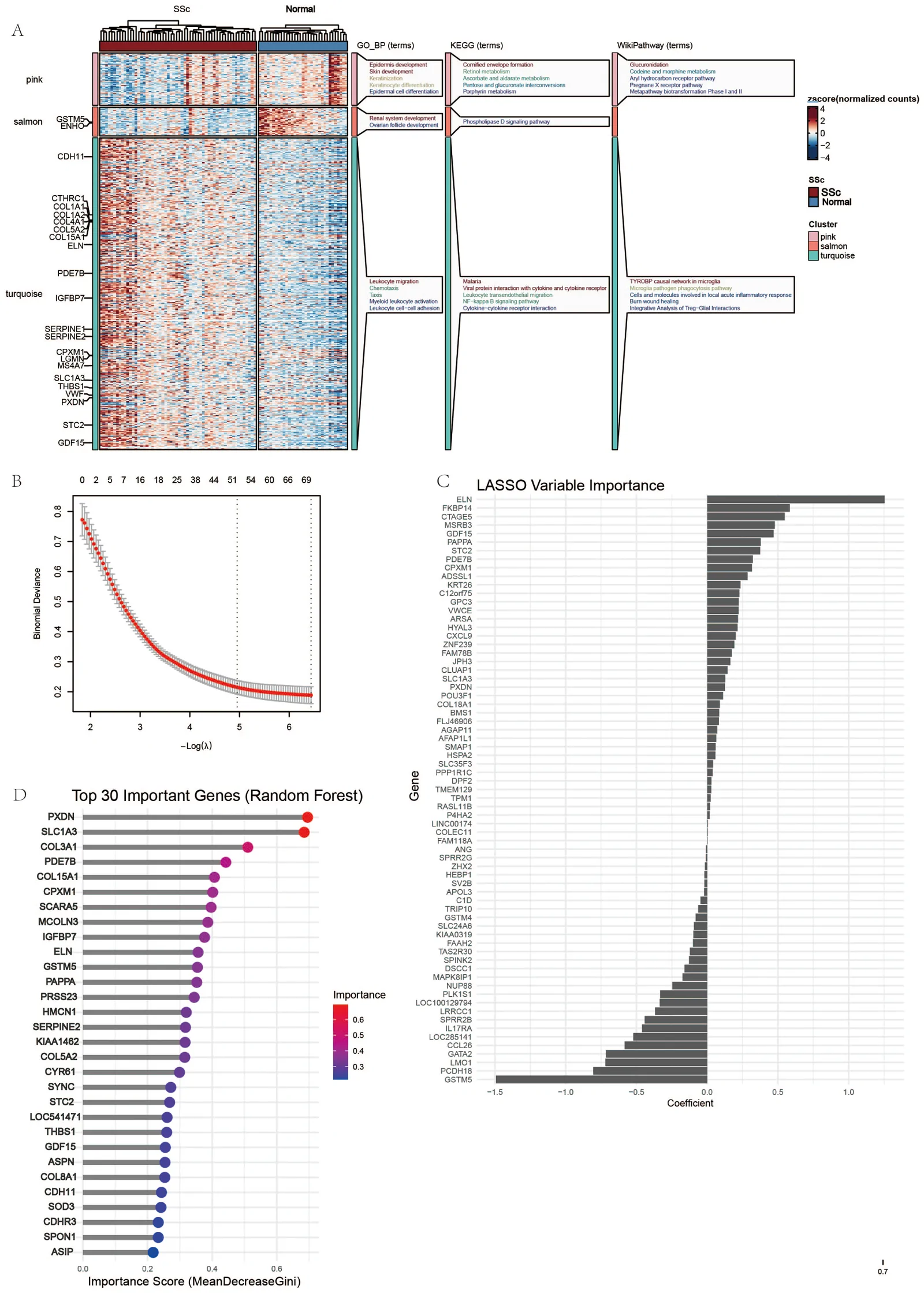
Machine learning–based identification of hub/diagnostic genes for systemic sclerosis (SSc). Three complementary machine-learning algorithms were applied to nominate genes associated with SSc status. **(A)** GO, KEGG and WikiPathway enrichment results for genes from WGCNA modules most strongly related to SSc. **(B, C)** LASSO regression identified 35 genes with non-zero coefficients; ELN showed the strongest positive association with SSc, while GSTM5 exhibited the strongest negative association. Notable positively associated genes include ELN, FKBP14, GDF15, and PDE7B; notable negatively associated genes include GSTM5 and GATA2. **(D)** Random forest analysis highlighted 42 highly important genes (Mean Decrease Gini > 50); top predictors included PXDN, SLC1A3, COL3A1, PDE7B, COL15A1, and CPXM1.

Finally, module–trait analysis identified MElightgreen as strongly positively correlated with the sex trait (**Figure 2G**). Pathway enrichment of MElightgreen revealed overrepresentation of processes related to sexual reproduction, including fertilization and male infertility (**Figure 3A**), supporting the biological relevance of the sex-associated signal.

### 3.2 Functional annotation reveals immune and fibrotic pathways in SSc-associated modules

To further characterize the biological functions of co-expression modules most strongly linked to SSc pathogenesis, we performed Gene Ontology (GO) enrichment analysis (**Figure 3A**). The top enriched biological processes (ranked by adjusted p-value) map directly onto core SSc pathology, including immune chemotaxis, leukocyte migration, leukocyte adhesion, myeloid cell activation, and formation of collagen-containing extracellular matrix (**Figure 3A**). These findings validate the disease relevance of the candidate modules and reinforce their involvement in the two central pathogenic axes of SSc: immune dysregulation and progressive fibrosis.

Comparative module analysis revealed a distinctive functional signature for the turquoise module. Genes in MEturquoise were most strongly enriched for immune response and chemotaxis pathways, distinguishing this module from others and highlighting immune-driven processes as a central transcriptional program in SSc. Together, these results implicate the turquoise module as a key mediator of the immune component of SSc pathogenesis and a likely contributor to subsequent fibrotic remodeling.

### 3.3 Multiple machine-learning algorithms converge on a core SSc gene signature

Guided by WGCNA-derived modules, we applied three complementary machine-learning methods to identify robust diagnostic features for SSc. LASSO regression selected 69 non-zero coefficient genes (**Supplementary Table 1**); among these *ELN* showed the strongest positive association with SSc status, while GSTM5 had the strongest negative association (**Figures 3B, C**). Other LASSO-positive genes of interest included *FKBP14, GDF15*, and *PDE7B*; notable negatively correlated genes included *GSTM5* and *GATA2*.

Random forest analysis nominated 77 highly important genes (Mean Decrease Gini > 0.1; **Supplementary Table 2**). Top predictors included *PXDN, SLC1A3, COL3A1, PDE7B, COL15A1*, and *CPXM1* (**Figure 3D**). SVM-RFE identified an optimal panel of 50 features (**Supplementary Table 3**). To synthesize these results, we applied a weighted scoring scheme that assigned each gene a score proportional to its selection frequency and importance across the three algorithms (score range 0–1), producing a consolidated, ranked gene list. The highest-ranked genes from this integrative approach were *PXDN, PDE7B, CPXM1, ELN*, and *GSTM5*.

**Figure 4:**
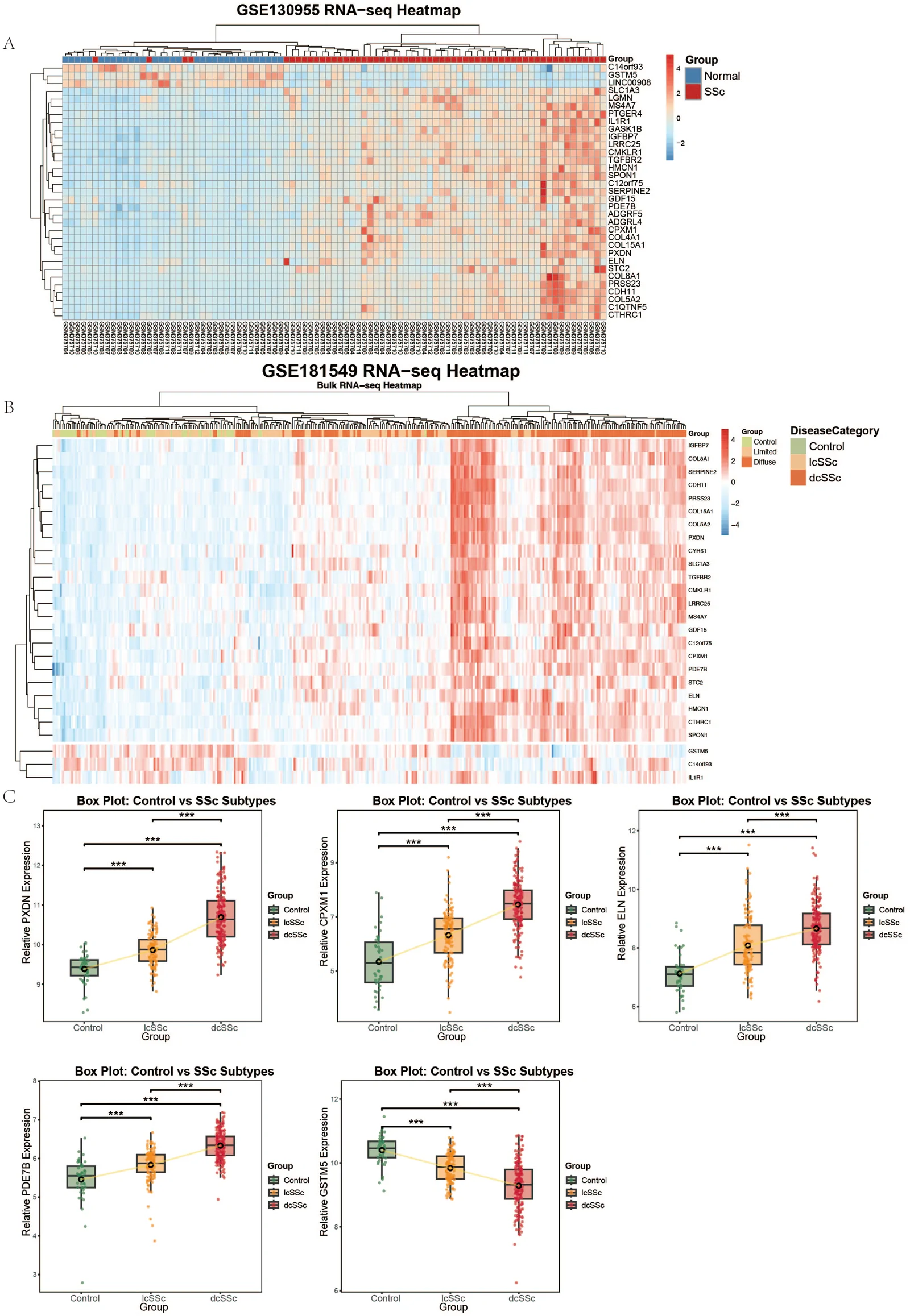
Expression profiles of machine-learning–selected key genes. **(A)** Heatmap of 33 key genes in GSE130955 showing distinct expression patterns between systemic sclerosis (SSc) patients and healthy controls. **(B)** Heatmap of 26 key genes in GSE181549 stratified by disease subtype, highlighting differential expression between limited cutaneous SSc (lcSSc) and diffuse cutaneous SSc (dcSSc). **(C)** Boxplots (or violin plots) of five representative genes (CPXM1, ELN, PXDN, PDE7B, GSTM5) in GSE181549 comparing expression across disease subtypes. ***p < 0.001.

A heatmap of the top 33 consolidated genes (**Figure 4A**) demonstrates clear separation between SSc and control samples. Several signature members have established profibrotic roles, supporting the biological validity of the panel: for example, *CTHRC1* is a marker of collagen-producing fibroblasts in lung, skin, and heart fibrosis, and *SERPINE2* (SerpinE2/protease nexin-1) — significantly upregulated in our cohort — has been implicated in cardiac fibrosis and *in vitro* fibrotic responses to AngII and TGF-β. Together, this multi-model pipeline distilled a concise, high-confidence gene signature from WGCNA modules and highlighted candidates (e.g., *PXDN, SERPINE2*) for further mechanistic study in SSc fibrosis.Validation of Feature Genes in Independent Corhorts and Their Clinical Relevance.

To validate the expression patterns of the key feature genes (*PXDN, PDE7B, CPXM1, ELN, GSTM5*) identified by our multi-odel approach, we analyzed two independent SSc transcriptomic datasets (GSE130955 and GSE181594). Unsupervised clustering based on these five genes reveals distinct expression profiles that effectively stratified samples by disease category (healthy control, lcSSc, dcSSc) (**Figure 4B**). Consistent with our primary findings, a detailed comparison of gene expression across groups demonstrated clear and graduated trends. Four genes, *ELN*, *PXDN*, *CPXM1*, and *PDE7B*, exhibited a progressive upregulation from healthy controls to lcSSc and further to dcSSc (**Figure 4C**). Conversely, *GSTM5* expression showed a stepwise downregulation across the same disease spectrum (**Figure 4C**). This expression gradient strongly suggests that these genes are not merely associated with SSc diagnosis but are quantitatively correlated with disease severity and clinical subtype, highlighting their potential as subtype-specific biomarkers.

### 3.4 Validation and diagnostic performance of feature genes for SSc subtype classification

To assess whether our feature-gene set can discriminate clinically relevant SSc subtypes, we trained a Random Forest classifier using disease subtype (lcSSc vs. dcSSc) as the outcome. Across repeated runs, *PDE7B*, *PXDN*, and *CPXM1* consistently ranked among the top predictors (**Supplementary Figures 1A–C**; **Supplementary Table 5**), demonstrating robust subtype-discriminatory capacity and implicating these genes as central markers of molecular differences between lcSSc and dcSSc.

**Figure 5:**
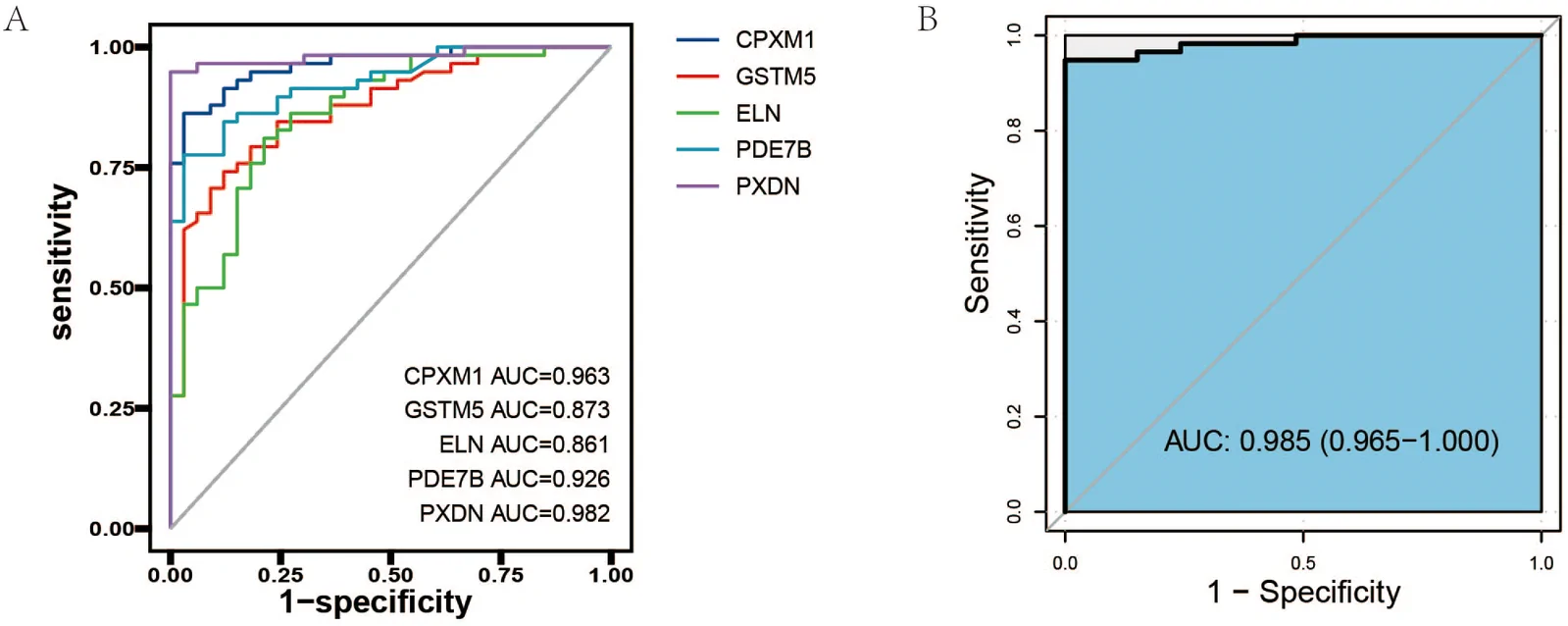
ROC validation of hub genes. **(A)** ROC curves for five candidate hub genes (*CPXM1, ELN, PXDN, PDE7B, GSTM5*) assessing discrimination between SSc patients and healthy controls. **(B)** ROC curve for a combined classifier integrating all five genes.

We next quantified diagnostic performance of five representative genes (*CPXM1, GSTM5, ELN, PDE7B, PXDN*) for distinguishing SSc patients from healthy controls by receiver operating characteristic (ROC) analysis (**Figure 5A**). All five genes showed strong predictive value: *PXDN* AUC = 0.982 (95% CI 0.957–1.000), *CPXM1* AUC = 0.963 (95% CI 0.931–0.996), PDE7B AUC = 0.926 (95% CI 0.876–0.976), *GSTM5* AUC = 0.873 (95% CI 0.801–0.946), and *ELN* AUC = 0.861 (95% CI 0.782–0.939). *PXDN*, *CPXM1*, and *PDE7B* in particular exhibited excellent discrimination (AUCs > 0.92). Combining all five genes into an integrated probability score further improved performance, yielding AUC = 0.985 (95% CI 0.965–1.000) (**Figure 5B**), indicating high potential for a multi-gene diagnostic panel.

To probe whether distinct clinical dimensions of SSc are driven by different molecular programs, we performed an analogous selection using disease duration as the phenotype. Top predictors of duration (e.g., *MARCH2*, *DPT*, *PLK2*; **Supplementary Figure 2**; **Supplementary Table 6**) showed minimal overlap with the five-gene diagnostic signature. This clear divergence suggests that the transcriptional determinants of long-term disease progression differ from those that define current disease state and subtype, underscoring the need for separate biomarker models for diagnosis/subtyping versus prognosis.

Additionally, we employed a Multilayer Perceptron (MLP) to predict key genes based on the GSE181549 dataset. The importance of genes was evaluated according to their corresponding weights and gradients. Notably, the five genes we had previously identified were ranked among the top features duration (**Supplementary Tables 12**, **13**).

### 3.5 Analysis of immune microenvironment infiltration associated with key genes

To interrogate immune dysregulation in SSc, we deconvoluted skin transcriptomes with CIBERSORT to estimate relative abundances of 22 immune cell types in SSc and control samples (**Supplementary Figure 3A**). Across cohorts, the dominant infiltrating populations were resting mast cells, M2 macrophages, resting CD4+ memory T cells, and resting dendritic cells. Comparative analysis revealed a significant enrichment of M2 macrophages in SSc lesions, consistent with their pro-fibrotic role (**Supplementary Figure 3B**). We also observed an increase in M1 macrophages, reflecting concurrent pro-inflammatory activity and indicating dynamic macrophage polarization in SSc.

To link these immune changes to our feature genes, we correlated gene expression with estimated immune cell proportions. *CPXM1, ELN, PXDN*, and *PDE7B* showed significant positive correlations with key SSc-enriched populations, notably M1 and M2 macrophages and activated CD4+ memory T cells (**Supplementary Figures 3C, D**). These associations were reproduced in independent cohorts: in the early-stage SSc cohort (GSE130955), *PXDN, CPXM1*, and *PDE7B* correlated strongly with M1 macrophage abundance (**Supplementary Figures 3E–G**), and the same three genes retained positive associations with M1 macrophages in the subtype-stratified cohort (GSE181549; **Supplementary Figures 3I–K**). In contrast, *GSTM5* displayed a significant negative correlation with M1 macrophages (**Supplementary Figure 3L**), aligning with its reduced expression in SSc (**Figure 4C**) and suggesting a potentially protective or anti-inflammatory role.

Together, these results connect the machine-learning–derived transcriptional signature to a remodeled immune microenvironment in SSc, particularly expansion of both pro-inflammatory (M1) and pro-fibrotic (M2) macrophage subsets. This linkage provides a plausible mechanistic route by which the identified hub genes may contribute to immune-driven fibrotic pathology in SSc.

### 3.6 Single-cell transcriptomic validation reveals cell type-specific expression of key biomarkers

**Figure 6:**
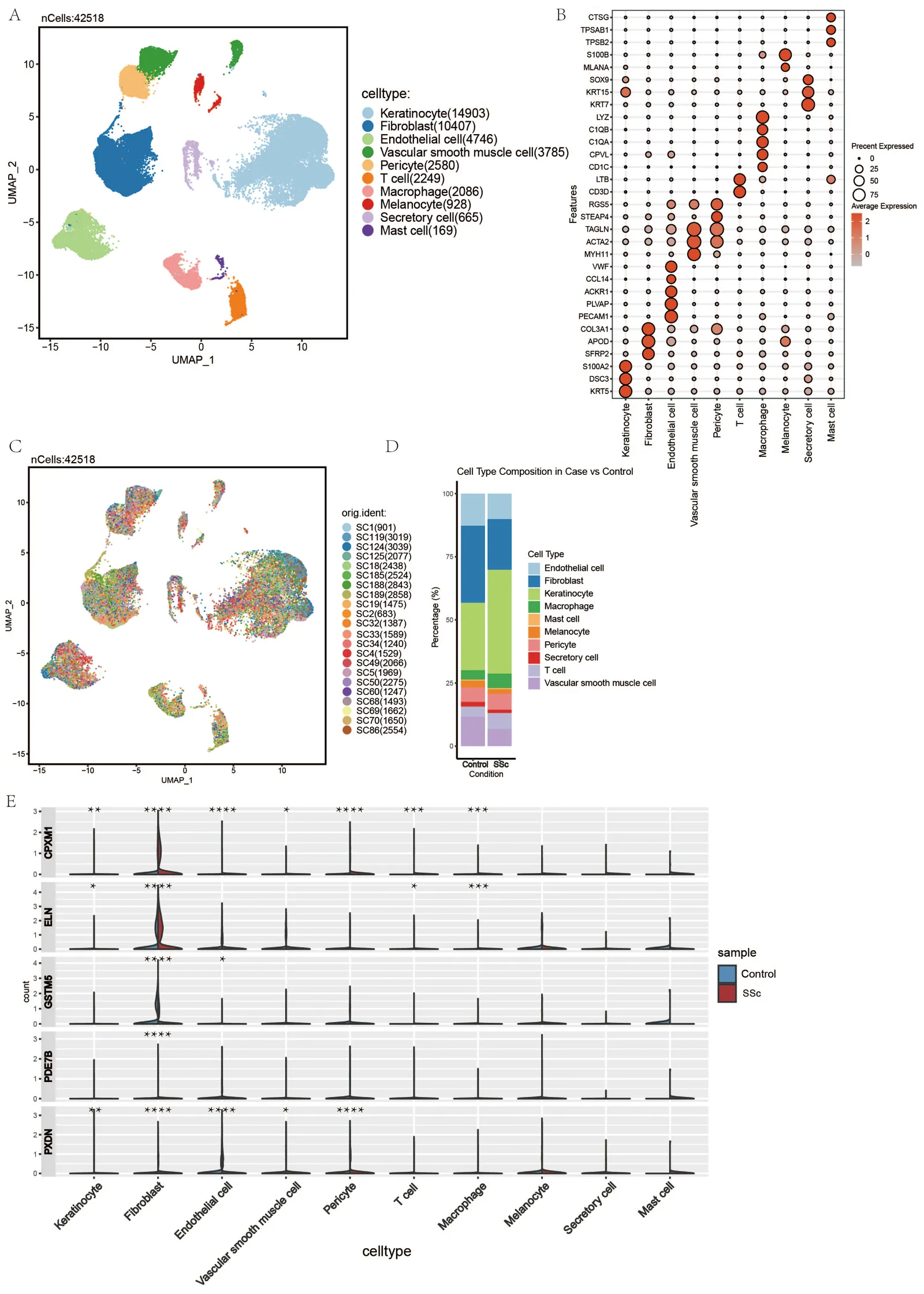
Single-cell RNA sequencing validation of hub gene expression. Single-cell characterization of hub gene expression in GSE138669. **(A)** UMAP projection showing annotated cell clusters identified in lesional skin. **(B)** Dot plot of canonical marker genes confirming cluster identities; dot size indicates fraction of cells expressing the marker and color intensity reflects mean expression. **(C)** Umap of the GSE138669 data to show the batch correction **(D)** Celltype composition of GSE138669 data. **(E)** Faceted violin plots showing cell type–specific expression distributions of the five hub genes (*CPXM1, ELN, PXDN, PDE7B, GSTM5*), highlighting enrichment or depletion across major cell populations. Significance levels are indicated as follows: *p < 0.05; **p < 0.01; ***p < 0.001; ****p < 0.0001.

To assign the machine-learning–derived biomarkers to specific cellular compartments, we analyzed scRNA-seq data from SSc lesional skin. Unsupervised clustering resolved major skin cell populations—fibroblasts, endothelial cells, keratinocytes, pericytes, vascular smooth muscle cells, and diverse immune cells (**Figures 6A–D**)—permitting precise mapping of candidate gene expression to discrete cell types.

This cell-resolved analysis revealed selective and functionally meaningful expression patterns for the five prioritized genes. Fibroblasts emerged as the principal cellular locus of dysregulation: *CPXM1* and *ELN* were markedly and specifically upregulated in SSc fibroblast populations relative to controls, and *PDE7B* showed similarly elevated, fibroblast-restricted expression (**Figure 6C**). *PXDN* was differentially expressed across multiple stromal and vascular compartments, including endothelial cells, fibroblasts, keratinocytes, pericytes, and vascular smooth muscle cells, suggesting broader roles in vascular and stromal remodeling. Conversely, *GSTM5* was substantially downregulated in SSc fibroblasts at single-cell resolution.

These cell type–specific signatures indicate non-redundant roles for the identified biomarkers and highlight fibroblasts as a central cellular nexus driving extracellular matrix deposition and remodeling in SSc.

### 3.7 Analysis of the high correlation between *PXDN* expression and ECM deposition in fibroblasts

Based on our literature research, peroxidasin (*PXDN*) contribute to fibrosis progression through collagen crosslinking (27). First, cells were stratified by *PXDN* expression into *PXDN*-high and *PXDN*-low groups within fibroblast and endothelial compartments (**Supplementary Figures 4A, E**). This produced 774 *PXDN*-high and 9,663 *PXDN*-low cells. SSc samples showed a markedly higher proportion of *PXDN*-high cells (11.6%) than controls (3.1%) in both fibroblast and endothelial populations (**Supplementary Figures 4B, F**), indicating disease-associated expansion of *PXDN*-expressing cells. Differential expression analysis of *PXDN*-high versus *PXDN*-low fibroblasts identified 1,002 upregulated and 90 downregulated genes (**Supplementary Figure 4C**). The upregulated program was strongly enriched for canonical profibrotic genes (*CTHRC1, COL1A1, COL3A1, THBS1, ELN, TNC, CTGF*) and GO terms related to extracellular matrix organization (**Supplementary Figure 4D**; **Supplementary Table 7**), linking high *PXDN* expression with an ECM-production phenotype. In endothelial cells, *PXDN*-high signatures included *CCND1, DLC1*, and *ID4* and were enriched for terms concerning regulation of cell migration (**Supplementary Figures 4G, H**; **Supplementary Table 8**).

Second, we performed an in silico *PXDN* knockdown using scTenifoldKnk on the fibroblast single-cell network to predict causal regulatory effects (20). Predicted perturbed genes included many core ECM and profibrotic factors (*COL1A1, COL3A1, COL1A2, SPARC, POSTN, ELN, TNC, COMP, SFRP2, CTHRC1*; **Supplementary Table 9**). The substantial overlap between genes elevated in *PXDN*-high fibroblasts and those predicted to respond to *PXDN* perturbation supports a central regulatory role for *PXDN* in driving a fibrotic transcriptional program.

Together, these orthogonal analyses indicate that *PXDN* marks an expanded fibroblast/endothelial subpopulation in SSc and is tightly linked to activation of ECM-producing, profibrotic networks, implicating *PXDN* as a potential mechanistic driver of matrix deposition in scleroderma.

### 3.8 Analysis of PXDN expression in correlation with fibrotic progression

**Figure 7:**
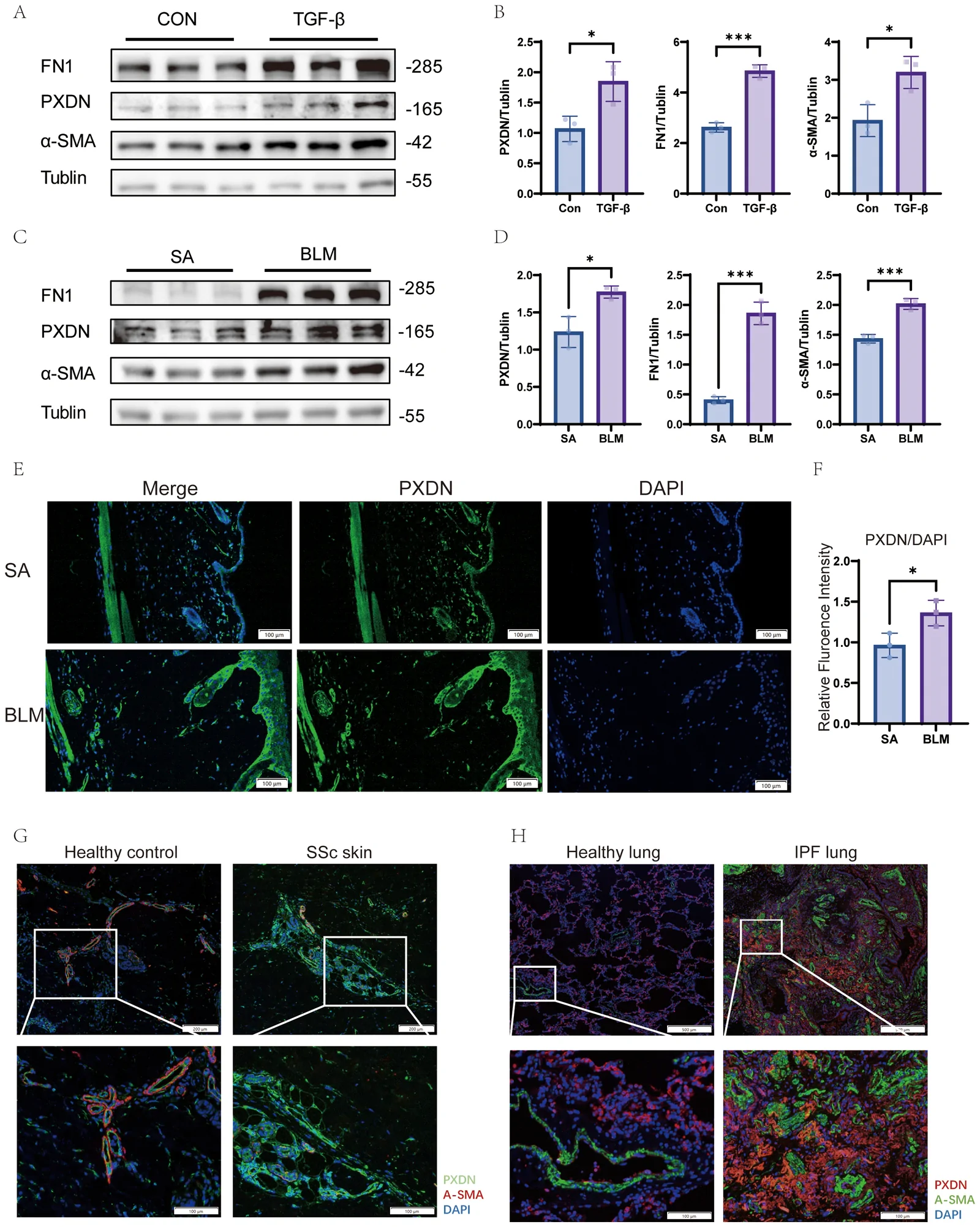
Experimental validation of PXDN in fibrosis. **(A)** Representative Western blots of PXDN, fibronectin-1 (FN1), and α-smooth muscle actin (α-SMA) in human foreskin fibroblasts (HFFs) treated ± TGF-β1 (10 ng/mL, 48 h). **(B)** Densitometric quantification of **(A)**; data are mean ± SEM from three independent experiments. *P < 0.05, ***P < 0.001 versus control (unpaired Student’s t-test). **(C)** Representative Western blots of PXDN, FN1, and α-SMA in lung tissues from mice treated with bleomycin (BLM) or saline control. **(D)** Densitometric quantification of **(C)**. **(E)** Immunofluorescence staining of PXDN in bleomycine induced skin fibrosis mouse model sections. **(F)** Fluorescence intensity quantification of E. **(G)** Immunofluorescence staining of PXDN in human skin and Scleroderma patient skin sections. **(H)** Immunofluorescence staining of PXDN in Healthy and IPF lungs.

To validate the association between *PXDN* and fibrosis, human foreskin fibroblasts (HFFs) were stimulated with recombinant TGF-β1 (10 ng/mL) for 48 hours. Western blotting of whole-cell lysates showed that TGF-β1 markedly increased PXDN protein levels alongside the canonical fibrotic marker fibronectin-1 (FN1) compared with untreated controls (**Figures 7A, B**), indicating PXDN is upregulated by TGF-β1 signaling in fibroblasts. Consistently, in a murine bleomycin (BLM) model of pulmonary fibrosis, lung tissues from BLM-treated mice exhibited pronounced upregulation of PXDN protein together with elevated FN1 and α-smooth muscle actin (α-SMA) relative to saline controls (**Figures 7C, D**).

Immunofluorescence analysis demonstrated significantly higher PXDN expression in both skin and lung tissue sections from patients with systemic sclerosis (SSc) and idiopathic pulmonary fibrosis (IPF) compared to healthy control tissues (**Figures 7G, H**). Furthermore, consistent with the human disease, a marked upregulation of PXDN was observed in the skin of mice following bleomycin-induced dermal fibrosis (**Figure 7E**). These *in vitro* and *in vivo* results support a positive association between PXDN expression and fibrotic progression, implicating PXDN as a potential contributor to fibrogenesis.

### 3.9 Screening for candidate therapeutics targeting hub genes

To identify repurposing opportunities for our five hub genes, we queried the Drug–Gene Interaction database (DGIdb). PDE7B returned four approved drugs — dyphylline, pentoxifylline, dipyridamole, and flavoxate hydrochloride — with indications spanning vasodilation, amyotrophic lateral sclerosis (ALS) support, and antithrombotic/anticoagulant uses, suggesting potential avenues for indication expansion (**Supplementary Table 10**). *ELN* also matched compounds in DGIdb, but these were not approved. No approved direct drugs were identified for *PXDN, CPXM1*, or *GSTM5*.

**Figure 8:**
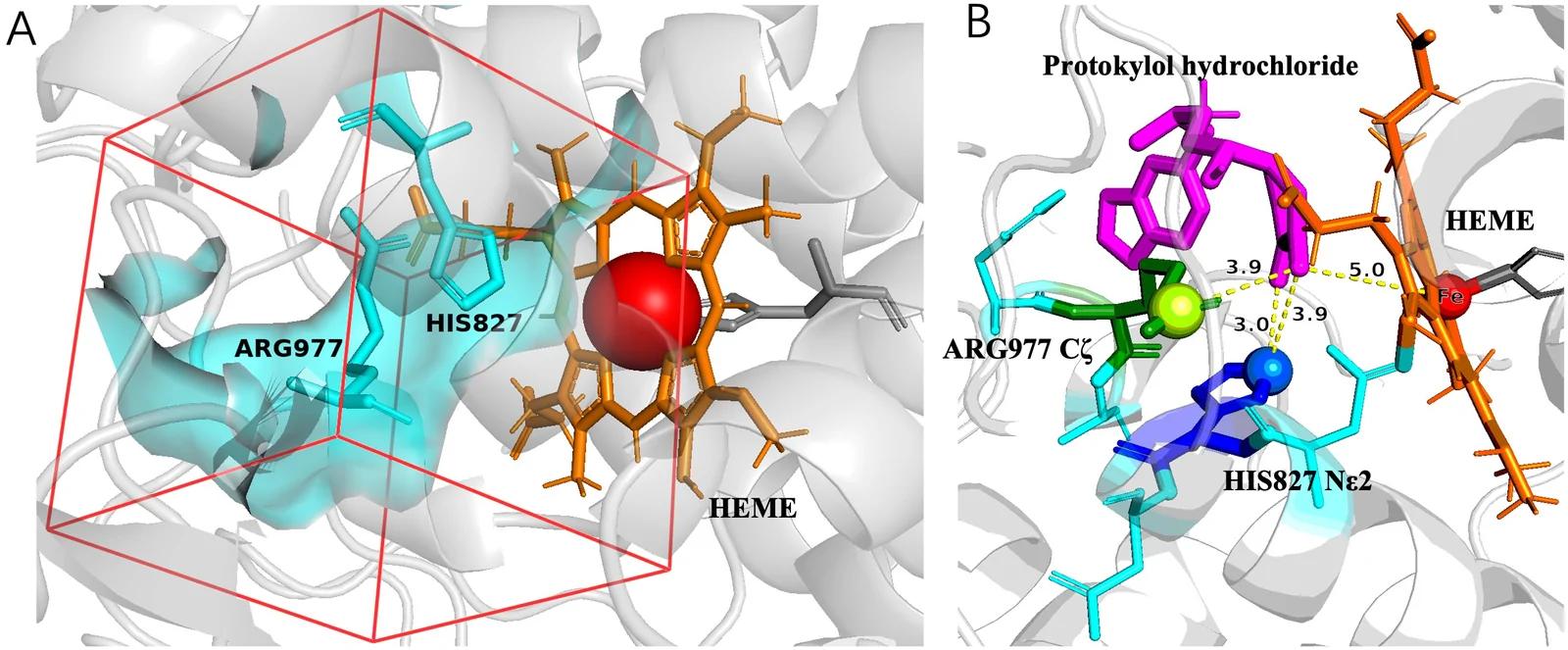
PXDN molecular docking results. **(A)** Definition of the PXDN distal docking pocket. The distal cavity (cyan mesh) is defined by the catalytic distal histidine (HIS827, blue sticks) and distal arginine (ARG977, blue sticks) together with surrounding cavity residues. The proximal histidine (HIS1074, gray sticks, excluded from the pocket) coordinates the heme iron (red sphere). The AutoDock Vina docking grid box (red, 12 × 12 × 12 Å) is centered on the midpoint between the catalytic Nϵ2 atom of HIS827 and the Cζ atom of ARG977, translated along the substrate-channel axis into the cavity interior. **(B)** The docking pose between Protokylol hydrochloride with PXDN. Protokylol hydrochloride binding pose within the distal cavity, with key contact distances to HIS827 Nϵ2 (3.0 Å) and ARG977 Cζ (3.9 Å).

To prospectively nominate PXDN-targeting agents, we performed in silico molecular docking of an FDA-approved small-molecule library against the PXDN structure. The FDA-approved drug library was docked. The top scorer by affinity, Belinostat (−8.11 kcal/mol), was excluded (Fe distance 2.0 Å, indicating direct metal coordination). Among 50 top-ranked ligands (321 poses), only Protokylol hydrochloride satisfied all three criteria: HIS827 Nϵ2, 3.0 Å; ARG977 Cζ, 3.9 Å; heme Fe, 5.0 Å; predicted affinity −7.66 kcal/mol (**Figure 8**). The pose lies between the His–Arg pair and the heme distal face, consistent with the canonical peroxidase inhibitor binding mode (23). Protokylol hydrochloride is the sole computational hit warranting experimental validation.

We emphasize that these docking results are strictly hypothesis-generating: absent biochemical or functional validation of PXDN inhibition, the proposed compound-PXDN interactions should be interpreted with caution and confirmed experimentally (e.g., SPR/ITC binding, enzymatic-activity, and cell-based assays) before any therapeutic claim.

## 4 Discussion

The identification of reliable biomarkers for systemic sclerosis (SSc) is significantly hampered by the disease’s pronounced molecular heterogeneity and the current absence of markers capable of robustly discriminating between its clinical subtypes. To address this gap, we implemented an integrated WGCNA-machine learning pipeline derive to compact, biologically informed biomarkers. This approach prioritized genes that are both central within disease-associated co-expression networks and demonstrate high predictive accuracy across independent cohorts, thereby mitigating spurious findings common to single-method analyses.

A central finding of this study is the identification of a robust five-gene signature (*PXDN, CPXM1, ELN, PDE7B, GSTM5*). This signature not only exhibits high diagnostic accuracy in discrimination of SSc from controls (AUC = 0.985) but also effectively differentiates the diffuse cutaneous (dcSSc) from the limited cutaneous (lcSSc) subtype. Functional enrichment analysis revealed that a Turquoise co-expression module strongly associated with SSc was enriched for extracellular matrix (ECM) remodeling and immune chemotaxis, reinforcing an immunofibrotic disease axis in SSc pathogenesis. Further single-cell data localized these genes primarily to fibroblasts and macrophages, supporting a cellular basis for their diagnostic value and implicating macrophage-fibroblast crosstalk as a core driver of clinical heterogeneity.

Within the signature, *ELN* and *PXDN* emerged as top-ranked diagnostic markers. *ELN* encodes elastin, a fundamental component of the extracellular matrix (ECM) that confers elasticity to tissues (28). While implicated in cardiac and pulmonary fibrosis (29), its specific role in SSc has been less defined. Our data associate elevated *ELN* expression with macrophage infiltration, suggesting that elastin remodeling contributes to the local inflammatory milieu and fibrotic remodeling in SSc skin. Similarly, *PXDN* (Peroxidasin), a peroxidase essential for collagen IV cross-linking (27), was significantly upregulated in SSc skin and showed a positive correlation with M1 macrophage proportions. Although *PXDN* was previously identified as a hub gene in an independent SSc cohort (30), our study validates its significance across subtypes using a complementary analytical framework. We also verified its high expression in fibroblasts and fibrotic model tissues. Its consistent involvement and link to pro-inflammatory macrophages underscore *PXDN* as a promising therapeutic target for mitigating fibrosis. Beyond collagen IV sulfilimine crosslinking and basement-membrane stabilization (16), PXDN acts at the ECM-immune interface: PXDN deficiency reprograms macrophages toward a pro-fibrolysis phenotype and promotes collagen resolution in liver fibrosis models (31). Although the organ context differs, this aligns with our immuno-fibrotic axis, and our data extend it to SSc skin, where PXDN marks an expanded fibroblast/vascular subset that tracks with ECM-producing programs and M1 macrophage abundance—strengthening our CIBERSORT- and single-cell-based findings, which rely on in silico deconvolution rather than direct immune profiling. The bleomycin model and TGF-β1 stimulation constitute correlative validation of PXDN induction, biologically plausible given PXDN’s TGF-β1-responsive expression and ECM-crosslinking function (15). We acknowledge that these data remain largely correlative and do not establish a causal role for PXDN in fibrosis progression, which will require dedicated loss- and gain-of-function studies.

Our analysis also uncovered novel insights into cyclic nucleotide signaling redox balance in SSc. *PDE7B*, a cAMP-specific phosphodiesterase, emerged as a key metabolic-related gene (32). Given that cAMP is a critical regulator of fibroblast activation, *PDE7B* may promote fibrosis via cAMP suppression, aligning with findings in idiopathic pulmonary fibrosis (IPF) (32) and cystic fibrosis (33). The inclusion of *GSTM5*, a gene involved in antioxidant defense, suggests that dysregulated redox-sensitive pathways contribute to disease progression, potentially enabling persistent fibroblast activation (34).

CIBERSORT immune deconvolution reinforced the centrality of immune-stromal interactions, showing strong correlations between signature genes and macrophage subsets (M1/M2). This aligns with a previous report noting a high prevalence of both M2 and M1 macrophage in SSc patients (18). The observations of elevated lymphocyte signatures in early-stage patients across studies further supports a dynamic immune contribution to disease progression.

*PXDN* emerged as a top candidate and was experimentally validated: TGF-β1 induced PXDN upregulation in human fibroblasts *in vitro*, and PXDN protein was significantly elevated in lung tissue from bleomycin-induced pulmonary fibrosis mice, implicating PXDN in matrix remodeling and fibrogenesis. To explore therapeutic relevance, we performed molecular docking (AutoDock Vina) against the PXDN model, protokylol emerged as the top-ranked hit against the PXDN distal cavity, but we present it as a proof-of-concept rather than a mature clinical candidate. Its value is methodological: in the docked pose it engages the distal cavity through specific hydrogen-bond and steric contacts with no direct heme-iron coordination, indicating that our screening strategy can prioritize genuine cavity binders over compounds relying on iron ligation alone—a common pitfall in peroxidase docking. We note, however, that the catechol moiety is a known peroxidase substrate and source of redox interference, so whether protokylol acts as a true inhibitor rather than a substrate must be confirmed experimentally. Structurally, protokylol also offers a tractable pharmacophore for optimization: future efforts will pursue bioisosteric replacement of the catechol to remove its metabolic and redox liabilities while retaining the aromatic core and hydrogen-bond donors, with SPR and enzymatic assays used to validate binding and inhibition.

Collectively, our integrative analysis defines a concise immuno-fibrotic signature with robust diagnostic and subtype discrimination and highlights PXDN as a mechanistically and therapeutically relevant nexus in SSc.

Despite the robust performance of our five-gene signature, this study has limitations. Our findings are derived from public transcriptomic datasets and in silico immune deconvolution; thus, experimental validation remains necessary. Key future directions include: 1) Longitudinal cohorts to assess prognostic and treatment-response value; 2) Functional studies (loss- and gain-of-function) in fibroblasts and macrophages and *in vivo* models to establish causality; and 3) Prospective validation across diverse cohorts and integration with other omics (proteomics, spatial transcriptomics) to enhance clinical applicability.

In conclusion, by integrating WGCNA with a multi-algorithm machine-learning strategy, we have derived a concise immunofibrotic five-gene signature that robustly distinguishes SSc and its major cutaneous subtypes. The signature emphasizes macrophage–fibroblast interplay and highlights perturbations in metabolic/redox pathway as central to disease heterogeneity. It provides validated molecular framework for patient stratification and prioritized targets for functional investigation and biomarker development of SSc.

## Data Availability

The datasets presented in this study can be found in online repositories. The names of the repository/repositories and accession number(s) can be found in the article/**Supplementary Material**.
