# Supplementary material for: A compact five-gene immuno-fibrotic profile distinguishing systemic sclerosis subtypes with PXDN validated as a fibrosis-associated target
Source: Front Immunol. 2026 Aug 19;17:1833652. doi: 10.3389/fimmu.2026.1833652 (PMC13534019; doi:10.3389/fimmu.2026.1833652)
Supplement: Supplementary file 1 [file Table1.docx]

Supplementary Material

# 1 Supplementary Figures and Tables

For more information on Supplementary Material and for details on the different file types accepted, please see [here](https://www.frontiersin.org/guidelines/author-guidelines#supplementary-material).

## 1.1 Supplementary Figures

**Supplementary Figure 1. Machine‑learning models for SSc subtype classification.** (A, B) Feature selection by LASSO regression for subtype discrimination, with ELANE and OPTN highlighted as prominent contributors. (C) Feature selection by Random Forest for subtype differentiation, identifying *ASPN, THBS1, PDE7B, PXDN,* and *CPXM1* as top predictors.

**Supplementary Figure 2.** **Machine Learning for identify key genes for disease duration.** (A, B) LASSO regression for disease duration related important genes, tops are TDRD6, MARCH2 and TCTEX1D2. (C) Random Forest for identify important genes in Scleroderma disease duration.

**Supplementary Figure 3. Immune landscape and gene–immune cell correlations in GSE130955 and GSE181549.** (A) Estimated proportions of 22 immune cell types across all samples inferred by CIBERSORT. (B) Comparison of immune cell infiltration between healthy controls and systemic sclerosis (SSc) patients; significant enrichments in SSc are indicated. (C, D) Spearman correlations between five key genes (CPXM1, ELN, PXDN, PDE7B, GSTM5) and immune cell fractions; positive and negative associations are shown. (E–G) Scatter plots showing the relationship between M1 macrophage proportion and expression of PXDN (E), PDE7B (F), and CPXM1 (G); fitted lines and correlation coefficients are shown. (H) Immune cell infiltration in GSE181549 dataset. (I–L) Scatter plots showing the correlation of PXDN (I), PDE7B (J), CPXM1 (K), and GSTM5 (L) with the proportion of M1 macrophages in GSE130955 dataset.

**Supplementary Figure 4.** **High-Low expression analysis in Fibroblast and Endothelial cell of PXDN gene.** (A) Violin plot comparing PXDN expression in fibroblast PXDN‑High versus PXDN‑Low clusters. (B) Bar plot showing the proportion of PXDN‑High fibroblasts in control versus SSc samples. (C) Volcano plot of differentially expressed genes between PXDN‑High and PXDN‑Low fibroblasts (log2FC on x‑axis, –log10 adjusted p on y‑axis). (D) GO enrichment analysis of genes upregulated in PXDN‑High fibroblasts, highlighting extracellular matrix and fibrotic pathways. (E)VlnPlot of Endothelial cell PXDN expression High or Low cluster. (F) Bar plot displaying the cell proportion of PXDN High group in Control and SSc. (G) Volcano plot of differential expression genes between PXDN-High and PXDN-Low cells. (H) GO Enrichment of Upregulated genes in PXDN-High/Low cell diffgene analysis.

## 1.2 Supplementary Tables
